# Supplementary material for: Stress deficits in reward behaviour are associated with and replicated by dysregulated amygdala-nucleus accumbens pathway function in mice
Source: Commun Biol. 2023 Apr 15;6:422. doi: 10.1038/s42003-023-04811-4 (PMC10105726; doi:10.1038/s42003-023-04811-4)
Supplement: Supplementary file 2 — Supplementary Information [file 42003_2023_4811_MOESM2_ESM.pdf]

Stress deficits in reward behaviour are associated with and replicated by dysregulated amygdala-nucleus accumbens pathway function in mice

Supplementary Information

9 Supplementary figures

2 Supplementary tables

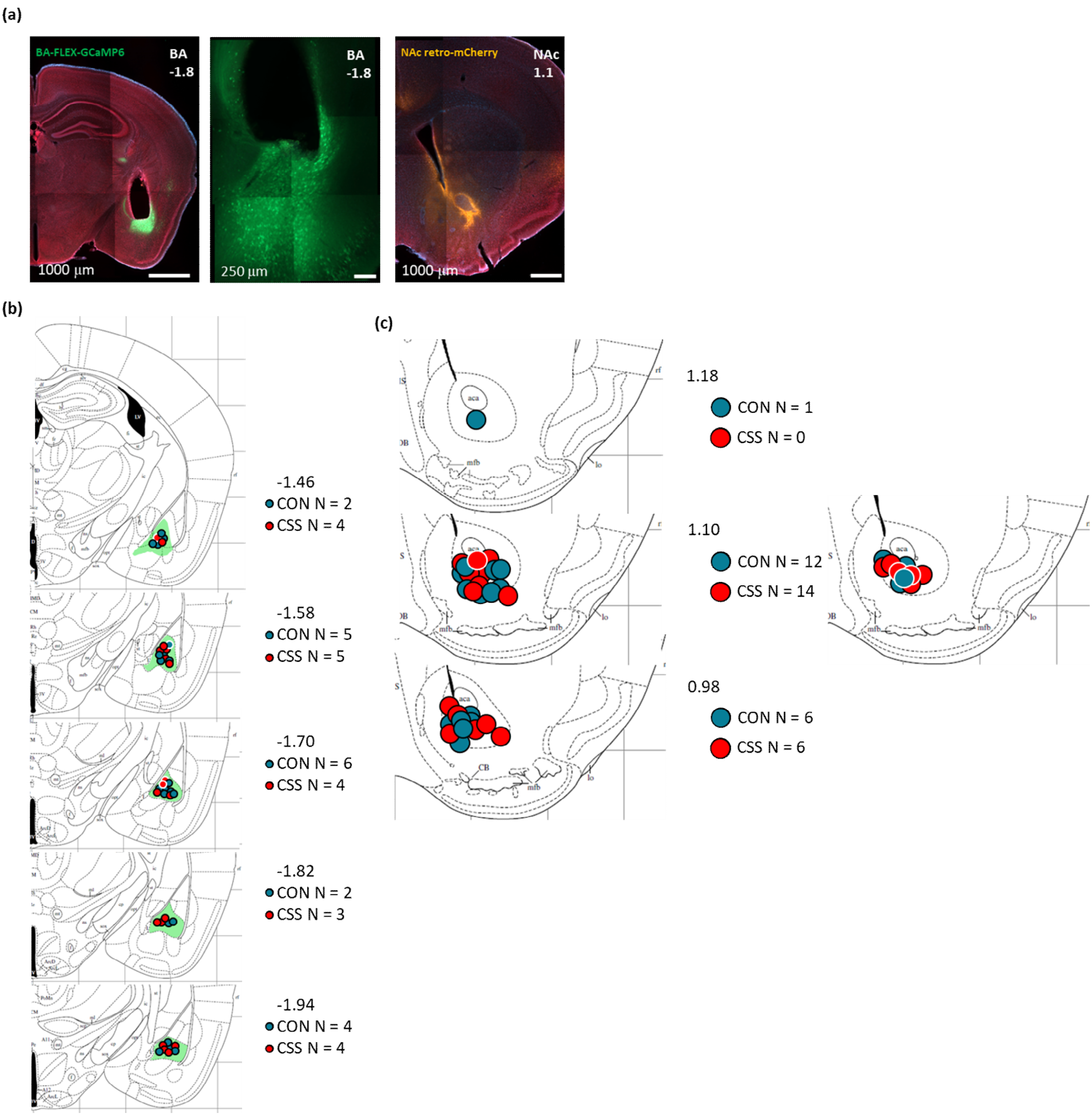

Supplementary Figure 1. Validation of AAV vector injection/expression and optic fibre placement in the experiment investigating chronic social stress and BA-NAc neuron activity during reward-directed behaviour
a. Representative microscope images (5x) of Nissl-stained coronal brain sections, showing: Left: the location of the optic fibre implant including its tip in the BA (-1.8 mm relative to bregma) of a control mouse and the colocalized AAV vector-expressed GCaMP6 fluorescence. Center: the same coronal brain section at higher magnification (20x). Right: NAc (1.1 mm relative to bregma) of a control mouse injected with retrograde AAV mCherry vector. Note that the 400 µm-diameter fibre implant projecting to the BA unavoidably causes some localised damaged of LA tissue, a major afferent to the BA. **b. c.** Schematics of coronal sections (Franklin & Paxinos, 2019) showing, respectively, the estimated BA location of the optic fibre tip and GCaMP6 expression and the estimated NAc location of mCherry expression, in CON and CSS mice, based on histological assessments; all mice that contributed fibre photometry and behavioural data are included. For illustration purposes all location estimates are depicted in the left hemisphere. Circles with white borders denote mice (CON n=1, CSS

n=3) for which for which fibre photometry data were collected for the DRLM test specifically, due to technical issues with these mice during the REV test. Images in b and c were used with permission of Elsevier, from The Mouse Brain Atlas, G. Paxinos & K.B.J. Franklin, 2nd edition, 2001; permission conveyed through Copyright Clearance Center, Inc.

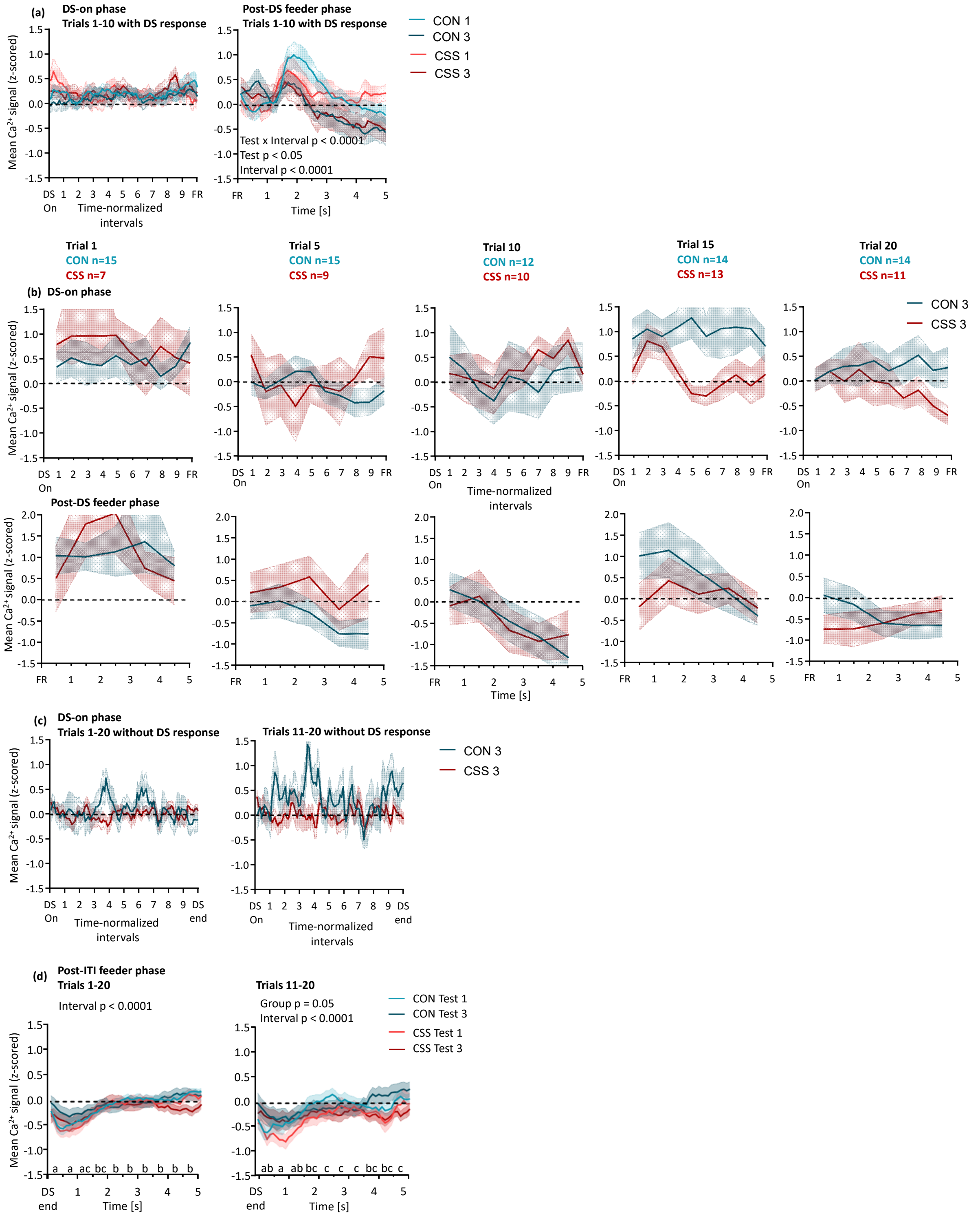

Supplementary Figure 2. Effects of chronic social stress on BA-NAc neural activity in the DRLM test

BA-NAc z-scored Ca^{2+} neural activity data are shown as group mean+S.E.M: **a.** Trials 1-10 with a DS response in tests 1 and 3, during the DS-on phase (left) and the post-DS feeder phase (right). **b.** Development of Ca^{2+} activity across individual trials 1, 5, 10, 15 and 20 in test 3. For each trial, the upper figure shows the DS-on phase and the lower figure the post-DS feeder phase, and the data include all mice that made a DS response at each specific trial. No statistical analysis was conducted. **c.** Trials 1-20 (left) and trials 11-20 (right) of test 3 without a DS response. Because there was no DS feeder response there is no post-DS feeder phase for these trials. **d.** Trials 1-20 (left) and trials 11-20 (right) inter-trial interval (ITI) feeder response phase, which started with a feeder response and had a duration of 5 s (subdivided into 0.5 s intervals). Statistical analysis was conducted using 2-

way (Group x Interval) or 3-way (Group x Test x Interval) mixed-model ANOVA. Test intervals indicated by different letters were significantly different in Tukey's multiple comparisons test: e.g. a vs b, a vs c, b vs c, $p < 0.05$ or lower.

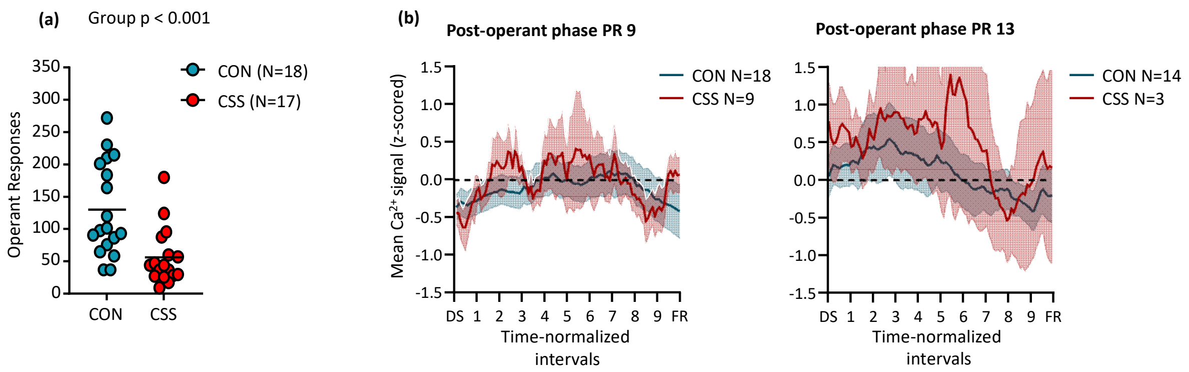

Supplementary Figure 3. Effects of chronic social stress on behaviour and BA-NAc neural activity in the REV test

a. At progressive ratio (PR) 5, number of operant responses; data are individual scores and group mean values, analysed using *t*-test. **b.** BA-NAc z-scored Ca^{2+} neural activity during the post-operant phase – time from DS onset to feeder response – at PR 9 (left) and PR 13 (right). No statistical analysis was conducted because of the relatively small sample size for CSS mice.

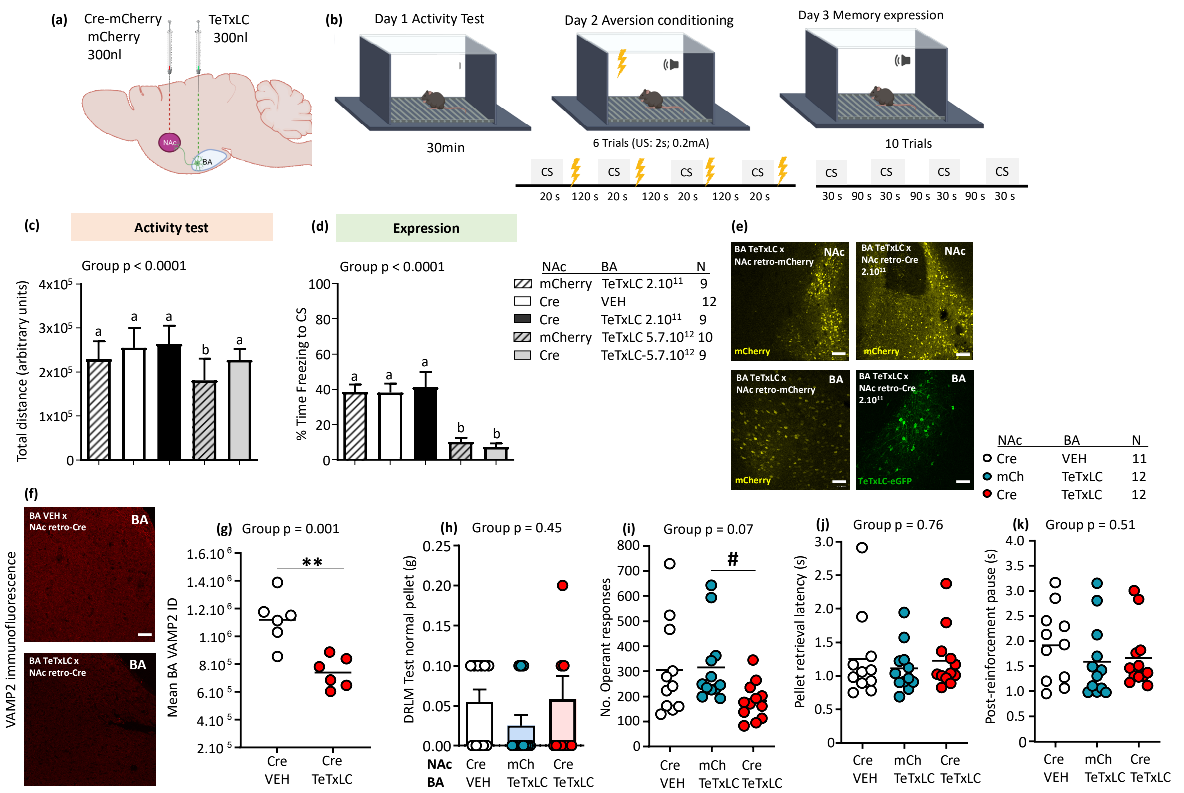

Supplementary Figure 4. Effects of tetanus-toxin-light-chain inhibition of BA-NAc neurons on reward-directed behaviour

a. Schematic showing bilateral injection sites of Cre-dependent AAV TeTxLC vector or vehicle in the BA and of retrograde AAV Cre vector or retrograde AAV mCherry vector in the NAc. **b-d.** Experimental design and data for the pilot study to establish the working titre for the AAV TeTxLC vector. **b.** Mice that received stereotaxic injections of AAV vectors underwent an activity test in the conditioning context on day 1, a tone-footshock (CS-US) conditioning test on day 2 (6 pairings of 20 s tone with sec 19-20 contiguous with 0.2 mA footshock), and a tone memory expression test on day 3 (10 x 30 s tone). **c.** In the activity test, total locomoor distance (mean+S.E.M.). **d.** In the tone expression test, % time freezing during presentation of the tone (mean+S.E.M.). **e.** Validation of the AAV vector system. Representative confocal micrographs (20x) of coronal brain sections showing: Left-upper: NAc of a control mouse injected with AAV mCherry vector. Left-lower: BA of a control mouse injected with AAV mCherry vector. Right-upper: NAc of an experimental mouse. Right-lower: BA of an experimental mouse. Scale bar = 50 µm. **f-g.** Validation of TeTxLC efficacy in terms of VAMP2 cleavage. **f.** Representative confocal micrographs (20x) of coronal brain sections with VAMP2 immuno-signal for control mouse (upper) and experimental mouse (lower). Scale bar = 50 µm. **g.** Integrated density values for VAMP2 immunostaining in BA coronal sections (individual and group means). **h.** DRLM test (individual values and group means): amount of normal diet consumed on test day 4. **i-k.** REV test (individual values and group means): **i.** Number of operant responses. **j.** Pellet retrieval latency. **k.** Post-reinforcement pause. Statistical analysis for the Pavlovian aversion learning-memory test was conducted using 2-way mixed-model ANOVA, for the VAMP2 data using an unpaired *t* test, and for the DRLM test and the REV test using 1-way ANOVA. Images a and b were created with BioRender.com.

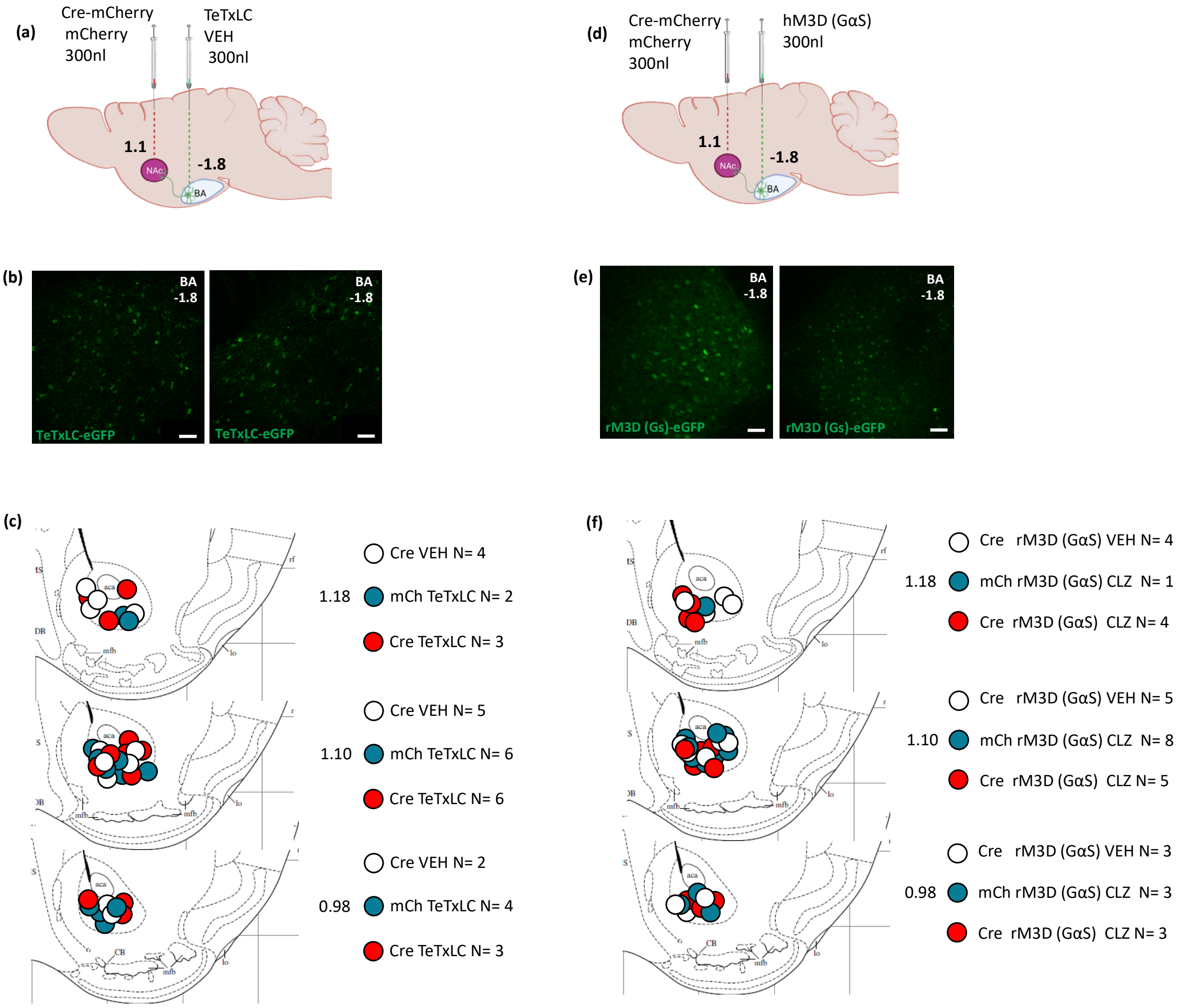

Supplementary Figure 5. Comparison of the distribution of eGFP-expressing BA-NAc neuronal cell bodies and NAc mCherry expression in the intermediate basal amygdala in the TeTxLC inhibition and DREADDs activation experiments.

a. Schematic showing bilateral injection sites (relative to bregma) of Cre-dependent TeTxLC AAV vector or vehicle in the BA and retrograde-Cre AAV vector or retrograde-mCherry AAV vector in the NAc. **b.** Representative confocal micrographs for BA with eGFP$^+$ neurons in an experimental mouse from the left hemisphere (left) and right hemisphere (right). (20x). **c.** Schematics of coronal sections (Franklin & Paxinos, 2019) showing the estimated NAc location of mCherry expression based on histological assessments. For illustration purposes all location estimates are depicted in the left hemisphere.

d. Schematic showing bilateral injection sites (relative to bregma) of Cre-dependent rM3D(Gs) AAV vector in the BA and retrograde-Cre AAV vector or retrograde mCherry AAV vector in the NAc. **e.** Representative confocal micrographs for BA with eGFP$^+$ neurons in an experimental mouse from the left hemisphere (left) and right hemisphere (right). **f.** Schematics of coronal sections (Franklin & Paxinos, 2019) showing the estimated NAc location of mCherry expression based on histological assessments. For illustration purposes all location estimates are depicted in the left hemisphere.

Magnification was 20x and scale bar = 50 µm.

Images in c and f were used with permission of Elsevier, from The Mouse Brain Atlas, G. Paxinos & K.B.J. Franklin, 2nd edition, 2001; permission conveyed through Copyright Clearance Center, Inc.

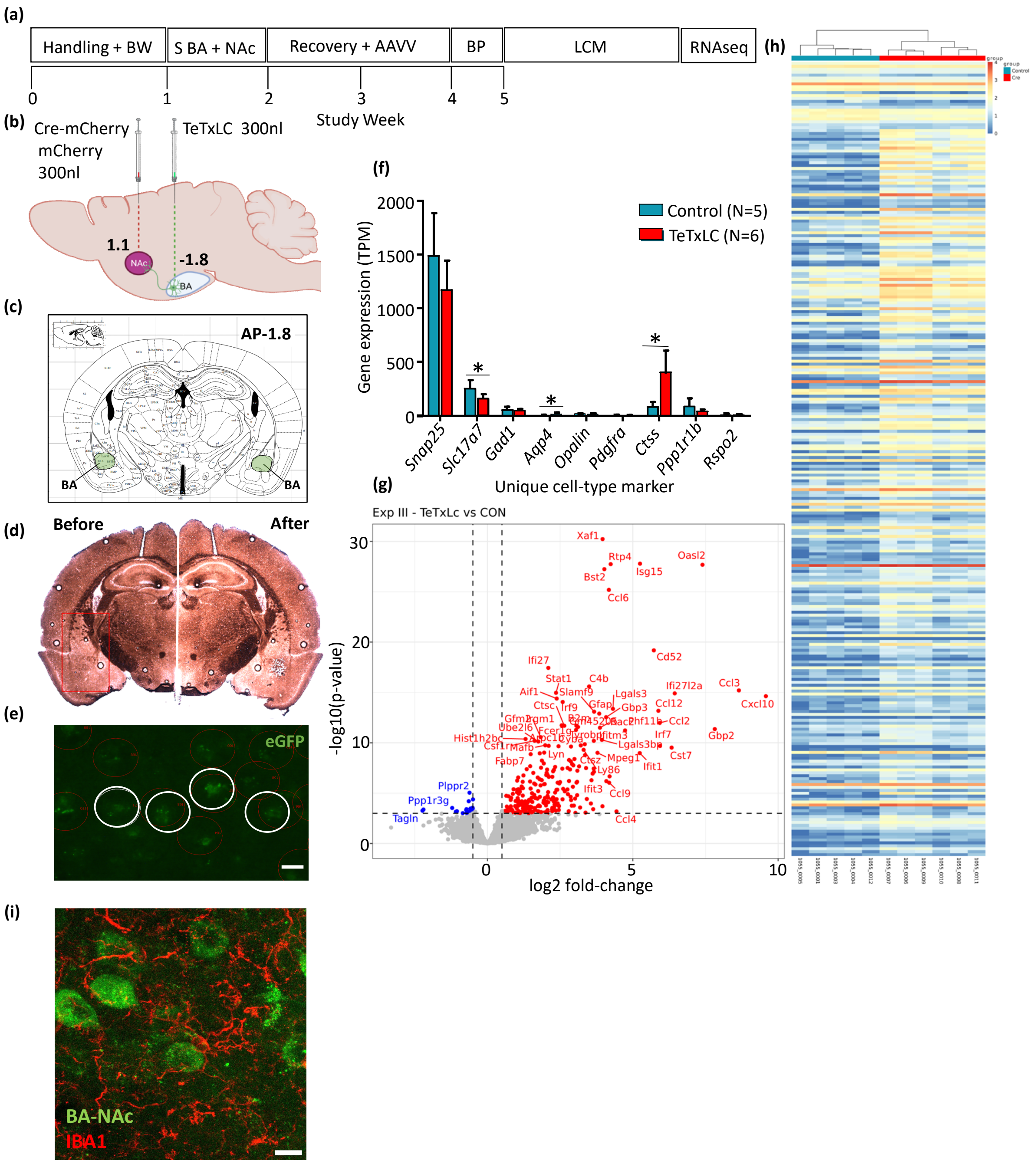

Supplementary Figure 6. Transcriptome-level validation of tetanus toxin light chain inhibition of BA-NAc neurons

a. Experimental design. Handling+BW: daily handling and measurement of body weight; S NAc+BA: stereotaxic surgery; Recovery+AAVV: recovery from surgery and AAV vector expression; BP: brains were PBS perfused; LCM: collection of BA-NAc CTB$^+$ labelled tissue using laser capture microdissection; RNAseq: RNA sequencing and differential gene differentiation analysis. **b.** Schematic showing bilateral injection site of Cre-dependent TeTxLC AAV vector in the BA and of retrograde Cre AAV vector or retrograde mCherry AAV vector in the NAc (relative to bregma), as used to generate 6 TeTxLC mice and 6 control mice; 1 control mouse was identified as an outlier by principal component analysis of RNA-seq data. **c.** Figure of coronal section from mouse brain atlas[35] at bregma level -1.8 mm with BA highlighted. **d.** Representative coronal image (5x) from brain of a TeTxLC mouse at bregma -1.8 mm before (left-hand) and after (right-hand) collection of eGFP$^+$ tissue using LCM. **e.** Representative coronal image (40x) from a TeTxLC mouse BA at bregma -1.8 mm. White circles indicate areas of eGFP$^+$ tissue demarcated for LCM. Scale bar = 200 µm. **f.** Expression levels (transcript per million; group mean+S.E.M.) of cell type-specific

marker genes: *Snap25* neuron, *Slc17a7* glutamate neuron, *Gad1* GABA neuron, *Aqp4,* astrocyte, *Opalin* myelinating oligodendrocyte, *Pdgfra* oligodendrocyte progenitor cell, *Ctss* microglia. *$p < 0.05$, unpaired *t* tests. **g.** Volcano plot for differential gene expression in TeTxLC compared with control mice: significantly up-regulated genes are shown in red and significantly down-regulated genes in blue. **h.** Heatmap representation of significantly differentially expressed genes depicting the complete separation of mice from the TeTxLC and control groups. **i.** Representative coronal image (63x) from brain of a mouse injected with CTB-555 in the BA at bregma -1.8 mm. BA-NAc neurons were labelled using CTB-555 and immunostaining for IBA1 (ionizing calcium-binding adaptor molecule 1) was used to identify microglia. For IBA1 immunostaining: primary antibody: 1:1000 rabbit anti-IBA1 (Wako #019-19741); secondary antibody: 1:500 donkey anti-rabbit 647 (ThermoFisher Scientific). Scale bar = 10 µm. Image b was created with BioRender.com. Image c was used with permission of Elsevier, from The Mouse Brain Atlas, G. Paxinos & K.B.J. Franklin, 2nd edition, 2001; permission conveyed through Copyright Clearance Center, Inc.

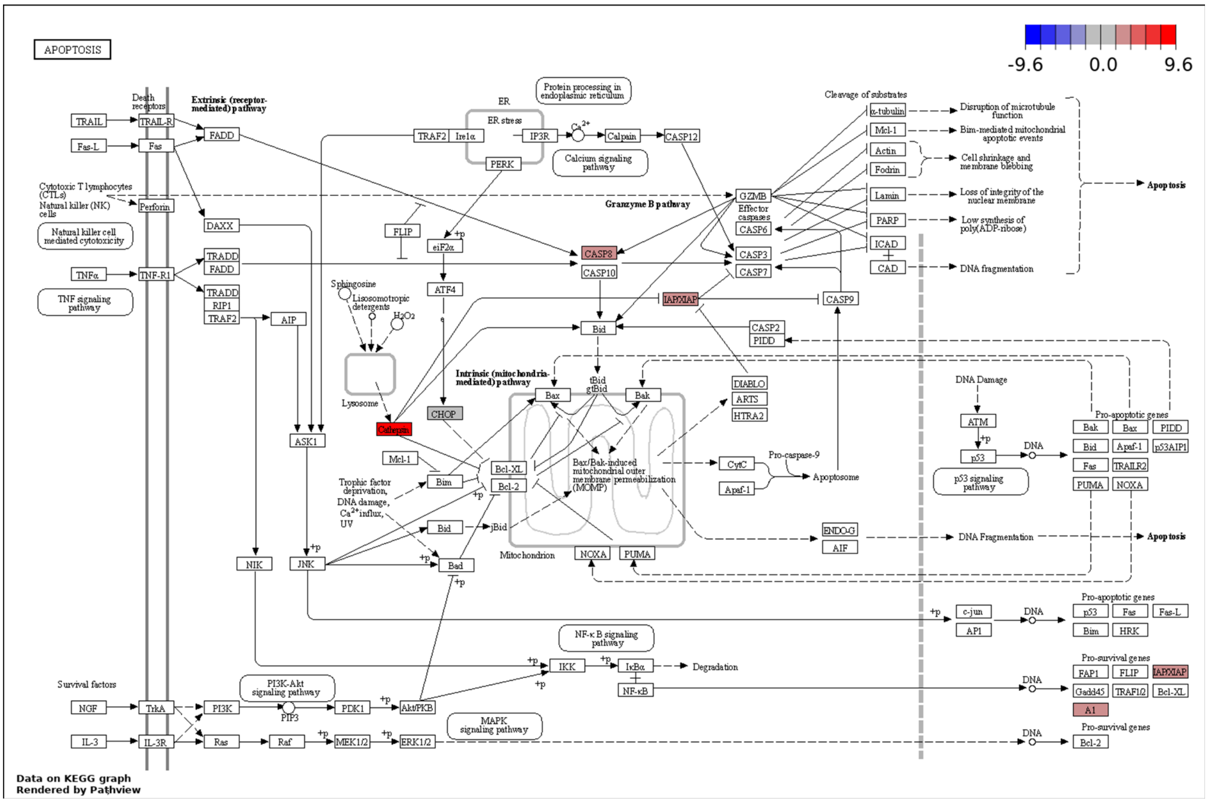

Supplementary Figure 7. Effects of tetanus-toxin-light-chain inhibition of BA-NAc neurons on their dysregulated expression of genes enriched in the KEGG apoptosis pathway

Gene hubs indicated in shades of red were significantly up-regulated in the transcriptome of BA-NAc neuron populations obtained from mice where these neurons had chronically expressed TeTxLc.

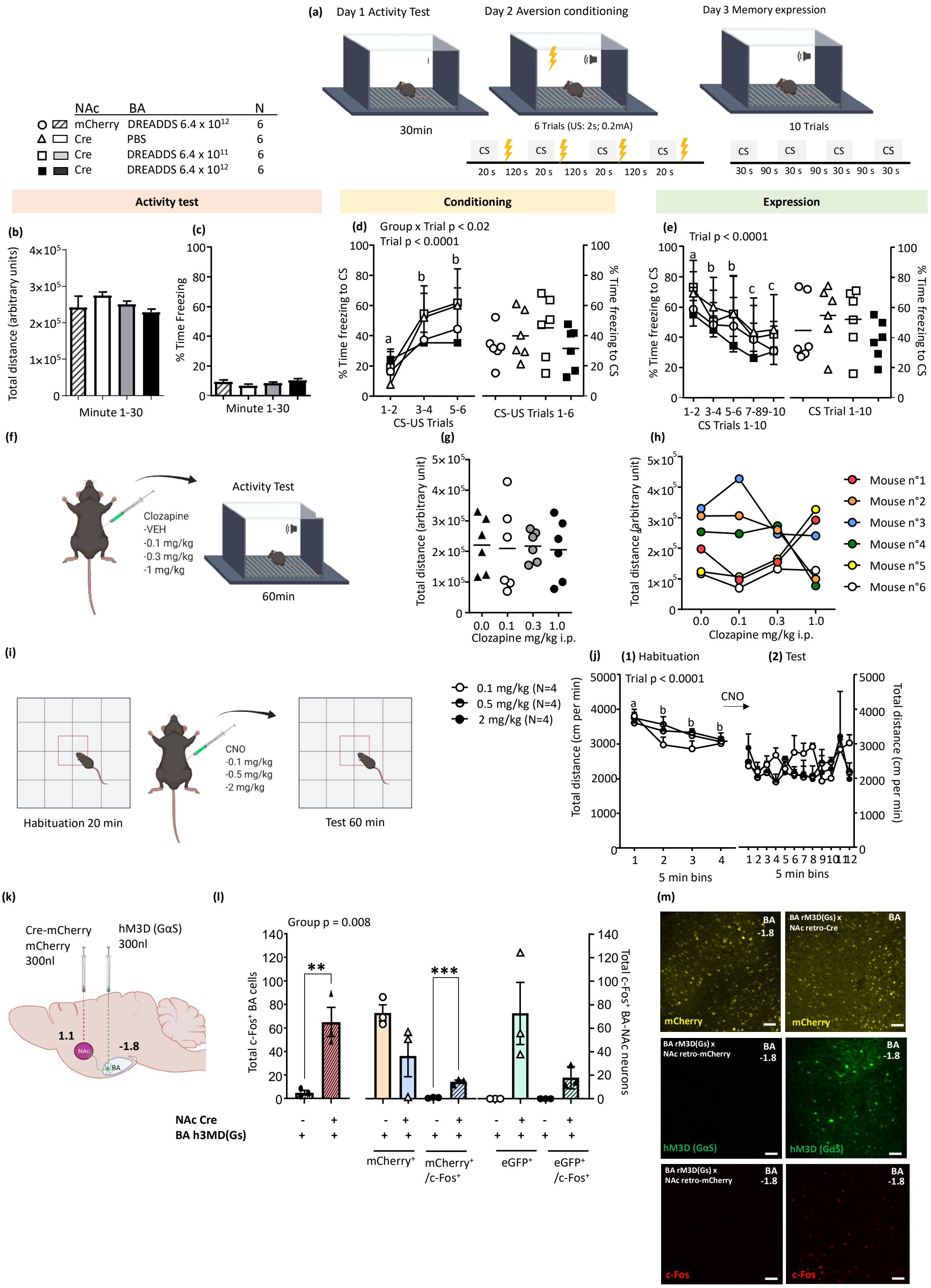

Supplementary Figure 8. Pilot experiments for effects of DREADDs activation of BA-NAc neurons on reward-directed behaviour

a-e. Experimental design and data for the pilot study to establish working titre for AAV DREADDs rM3D(Gs) vector. **a.** Mice that had received stereotaxic injections of AAV vectors underwent an activity test in the conditioning context on day 1, a tone-footshock (CS-US) conditioning test on day 2 (6 pairings of 20 s tone with

sec 19-20 contiguous with 0.2 mA footshock), and a tone memory expression test on day 3 (10 x 30 s tone). **b.** Activity test, total locomoor distance (group mean+S.E.M.). **c.** Activity test, % time spent freezing (group mean+S.E.M.). **d.** Pavlovian conditioning test, % time freezing during presentation of the tone for trials 1-2, 3-4, 5-6 and for trial 1-6. **e.** Tone-CS memory expression test, % time freezing during presentation of the tone for trials 1-2, 3-4, 5-6, 7-8, 9-10 and for trial 1-10. **f-h.** Experimental design and data for the pilot study to establish a clozapine dose with minimal locomotor effects. **f.** Mice without AAV vectors were injected with VEH only or clozapine before undergoing an activity test in a conditioning chamber. **g-h.** Activity test locomotion: **g.** scatter plots and **h.** line plots for individual mice. **i-j.** Experimental design and data for the pilot study to establish a clozapine-N-oxide dose with minimal locomotor effects. **i.** Mice that had received stereotaxic injections of AAV vectors were placed in an open field for 20 min habituation prior to being injected with CNO and returned to the open field for 60 min test. **j.** Open field locomotion during the habituation and test phases. **k-m.** Schematic showing bilateral injection sites of Cre-dependent AAV rM3D(Gs) vector in the BA and of retrograde AAV Cre vector or retrograde AAV mCherry vector in the NAc. **l-m.** Mice were injected with clozapine and after 105 min were perfused; coronal brain sections underwent c-Fos immunostaining. **l.** Comparison of number of c-Fos^{+} cells in the BA of rM3D(Gs) mice (N=3) and control mice (N=3), in terms of total number, number of m-Cherry^{+} BA-NAc neurons and eGFP^{+} BA-NAc neurons. **m.** Representative confocal micrographs (20x) of coronal brain sections showing: Left-upper: BA with mCherry in a control mouse. Left-middle: BA lacking eGFP in a control mouse. Left-lower: BA with c-Fos signal in a control mouse. Right-upper: BA with mCherry in a rM3D(Gs) mouse. Right-middle: BA with eGFP in a rM3D(Gs) mouse. Right-lower: BA with c-Fos signal in a rM3D(Gs) mouse. Scale bar = 50 µm. Statistical analysis for the Pavlovian aversion learning-memory test was conducted using 2-way mixed-model ANOVA, for the activity test x clozapine experiment using 1-way ANOVA and for the open field test x CNO experiments using 2-way mixed-model ANOVA. Analysis of the c-Fos data was conducted using unpaired *t* test. Images in a, f, I and k were created with BioRender.com.

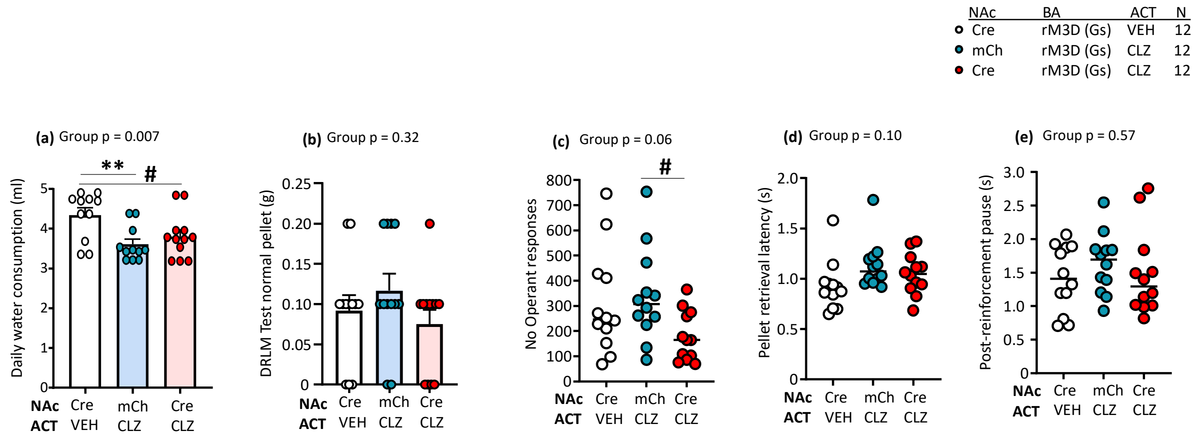

Supplementary Figure 9. Effects of DREADDs activation of BA-NAc neurons on reward-directed behaviour
a. Volume of water consumed per day during the period of CNO dosing via the drinking water. **b.** DRLM test: amount of normal diet consumed on test day 4. **c-e.** REV test: **c.** Number of operant responses. **d.** Pellet retrieval latency. **e.** Post-reinforcement pause. Statistical analysis of data for water consumption, DRLM test and REV test was in each case conducted using 1-way ANOVA.

Table S1. Details of mouse body weight and food intake at baseline, re-baseline and during behavioural testing

Expt	Group	Baseline		Re-Baseline		Testing		
		BW (g)	Food (g)	BW (g)	Food (g)	% re-BBW %	%re-B Food	Food (g)
CSS	CON (14)	29.1±1.6	3.6±0.3	29.5±1.6	3.6±0.4	98.3±1.4	81.4±5.0	2.9±0.3
	**CSS** (14)	30.1±1.2	3.7±0.2	30.5±1.1	4.8±0.5****	98.7±1.2	87.8±10.3*	4.2±0.5****
CSS- Photometry	CON (21)	28.7±1.5	3.6±0.3	29.7±1.6	4.1±0.4	98.8±2.7	86.9±11.7	3.6±0.4
	**CSS** (21)	28.7±1.5	3.5±0.4	30.4±1.7	5.5±0.7****	99.2±1.8	87.0±4.9	4.8±0.6****
BA-NAc TeTxLC inhibition	Cre x VEH (12)	28.7±2.0	3.5±0.4	28.3±2.1	3.7±0.4	95.7±3.4	111.3±9.6	4.2±0.5
	mCh x TeTxLC (12)	28.6±2.0	3.5±0.6	28.4±2.6	3.6±0.5	94.6±4.6	105.3±9.1	3.8±0.7
	**Cre x TeTxLC** (12)	29.3±1.8	3.8±0.4	28.9±1.8	3.9±0.5	95.7±4.6	107.9±8.7	4.2±0.8
BA-NAc DREADDs activation	Cre x VEH (12)	25.5±1.2	3.6±0.4	28.4±1.1	3.6±0.3	95.0±3.0$^{\#}$	85.7±22.7$^{\#}$	3.0±0.7$^{\#\#}$
	mCh x CLZ (12)	25.4±1.3	3.4±0.4	28.2±1.2	3.3±0.3	98.9±2.8	67.6±9.2	2.2±0.2
	**Cre x CLZ** (12)	25.9±1.6	3.6±0.3	28.7±1.7	3.6±0.3	97.3±3.5	88.9±15.4*	3.2±0.6***

Values are mean±SD

CSS: **CSS** > CON, * p<0.05, **** p<0.0001, unpaired t-tests

CSS-Photometry: **CSS** > CON, **** p<0.0001, unpaired t-tests

BA-NAc DREADDs: **Cre x CLZ** > mCh x CLZ * p<0.05, ***p<0.001, 1-way ANOVA followed by Tukey's post hoc test

Cre x VEH > mCh x CLZ $^{\#}$ p<0.05, $^{\#\#}$ p<0.01, 1-way ANOVA followed by Tukey's post hoc test

Table S2. Total DS-US trials with a response in each discriminative reward learning-memory test

Expt	Group	Test 1	Test 2	Test 3	Test 4
CSS	CON (14)	23.4±7.7	24.0±5.0	25.9±5.1	
40 trials/test	**CSS** (13)	16.9±4.1	19.2±7.2	19.0±7.9**	
CSS-Photometry	CON (19)	25.3±5.5	26.8±5.2	26.6±4.4	
31 trials/test	**CSS** (20)	18.7±7.9	18.7±6.8	17.8±7.3***	
BA-NAc TeTxLC	Cre x VEH (11)	30.3±5.4	30.9±7.2	31.4±7.5	30.0±7.8
inhibition	mCh x TeTxLC (12)	28.7±4.2	31.3±4.7	30.7±6.1	32.9±4.9
40 trials/test	**Cre x TeTxLC** (12)	26.1±6.2	26.9±6.8	28.5±7.3	29.1±6.4
BA-NAc DREADDs	Cre x VEH (12)	23.8±9.3	28.7±7.3	30.2±8.1	30.8±8.7
activation	mCh x CLZ (12)	23.8±7.4	28.9±6.3	31.7±6.8	31.0±5.2
40 trials/test	**Cre x CLZ** (12)	26.7±9.4	24.2±6.3	26.5±8.5	25.8±7.5

Values are mean±SD
CSS: **CSS** < CON, ** p<0.006, 1-way repeated measures ANOVA
CSS-Photometry: **CSS** < CON, **** p<0.0001, 1-way repeated measures ANOVA
