# Supplementary material for: Stress deficits in reward behaviour are associated with and replicated by dysregulated amygdala-nucleus accumbens pathway function in mice
Source: Commun Biol. 2023 Apr 15;6:422. doi: 10.1038/s42003-023-04811-4 (PMC10105726; doi:10.1038/s42003-023-04811-4)
Supplement: Supplementary file 3 — Reporting Summary [file 42003_2023_4811_MOESM3_ESM.pdf]

nature portfolio

Corresponding author(s): Pryce, C.R.

Last updated by author(s): 17.03.2023

Reporting Summary

Nature Portfolio wishes to improve the reproducibility of the work that we publish. This form provides structure for consistency and transparency in reporting. For further information on Nature Portfolio policies, see our Editorial Policies and the Editorial Policy Checklist.

Statistics

For all statistical analyses, confirm that the following items are present in the figure legend, table legend, main text, or Methods section.

n/a	Confirmed	
☐	☒	The exact sample size (*n*) for each experimental group/condition, given as a discrete number and unit of measurement
☐	☒	A statement on whether measurements were taken from distinct samples or whether the same sample was measured repeatedly
☐	☒	The statistical test(s) used AND whether they are one- or two-sided<br>*Only common tests should be described solely by name; describe more complex techniques in the Methods section.*
☒	☐	A description of all covariates tested
☐	☒	A description of any assumptions or corrections, such as tests of normality and adjustment for multiple comparisons
☐	☒	A full description of the statistical parameters including central tendency (e.g. means) or other basic estimates (e.g. regression coefficient) AND variation (e.g. standard deviation) or associated estimates of uncertainty (e.g. confidence intervals)
☐	☒	For null hypothesis testing, the test statistic (e.g. *F*, *t*, *r*) with confidence intervals, effect sizes, degrees of freedom and *P* value noted<br>*Give P values as exact values whenever suitable.*
☒	☐	For Bayesian analysis, information on the choice of priors and Markov chain Monte Carlo settings
☒	☐	For hierarchical and complex designs, identification of the appropriate level for tests and full reporting of outcomes
☒	☐	Estimates of effect sizes (e.g. Cohen's *d*, Pearson's *r*), indicating how they were calculated

Our web collection on statistics for biologists contains articles on many of the points above.

Software and code

Policy information about availability of computer code

Data collection	IntelliMaze (TSE Systems)<br>LabVIEW (National Instruments)<br>MATLAB (MathWorks)<br>CellTools (MMI)
Data analysis	MATLAB (MathWorks)<br>GraphPad (v. 9, Prism)<br>SPSS (v. 26, IBM)<br>RSEM (v. 1.3.0)<br>featureCounts (v. 1.5.1)<br>R (v. 4.1.0)<br>Bioconductor (v. 3.12)<br>mclust (v. 5.4.7)<br>DESeq2 (v. 1.32.0)<br>PCAtools (v. 2.4.0)<br>enrichR (v. 3.0)

For manuscripts utilizing custom algorithms or software that are central to the research but not yet described in published literature, software must be made available to editors and reviewers. We strongly encourage code deposition in a community repository (e.g. GitHub). See the Nature Portfolio guidelines for submitting code & software for further information.

Data

Policy information about availability of data

All manuscripts must include a data availability statement. This statement should provide the following information, where applicable:

- Accession codes, unique identifiers, or web links for publicly available datasets
- A description of any restrictions on data availability
- For clinical datasets or third party data, please ensure that the statement adheres to our policy

Raw sequencing data and gene expression matrices from the CSS - BA-NAc neuron transcriptome and BA-NAc neuron tetanus toxin transcriptome experiments were deposited in the Gene Expression Omnibus and can be accessed with accession codes GSE216587 and GSE216588, respectively. The code that was used to process and analyse the expression data is available on https://github.com/platrad-uzh/CSS_effects_on_BA-NAc_rnaseq.

Human research participants

Policy information about studies involving human research participants and Sex and Gender in Research.

Reporting on sex and gender	*Use the terms sex (biological attribute) and gender (shaped by social and cultural circumstances) carefully in order to avoid confusing both terms. Indicate if findings apply to only one sex or gender; describe whether sex and gender were considered in study design whether sex and/or gender was determined based on self-reporting or assigned and methods used. Provide in the source data disaggregated sex and gender data where this information has been collected, and consent has been obtained for sharing of individual-level data; provide overall numbers in this Reporting Summary. Please state if this information has not been collected. Report sex- and gender-based analyses where performed, justify reasons for lack of sex- and gender-based analysis.*
Population characteristics	*Describe the covariate-relevant population characteristics of the human research participants (e.g. age, genotypic information, past and current diagnosis and treatment categories). If you filled out the behavioural & social sciences study design questions and have nothing to add here, write "See above."*
Recruitment	*Describe how participants were recruited. Outline any potential self-selection bias or other biases that may be present and how these are likely to impact results.*
Ethics oversight	*Identify the organization(s) that approved the study protocol.*

Note that full information on the approval of the study protocol must also be provided in the manuscript.

Field-specific reporting

Please select the one below that is the best fit for your research. If you are not sure, read the appropriate sections before making your selection.

☒ Life sciences ☐ Behavioural & social sciences ☐ Ecological, evolutionary & environmental sciences

For a reference copy of the document with all sections, see nature.com/documents/nr-reporting-summary-flat.pdf

Life sciences study design

All studies must disclose on these points even when the disclosure is negative.

Sample size	With the treatment, chronic social stress (CSS), a sample size of 12-15 per CSS and control group yields reproducible, statistically significant effects on behavioural, physiological and neurobiological measures. This sample size was therefore used for CSS behavioural experiments in this study. The same sample size was also used for the experiments investigating effects of basal amygdala-nucleus accumbens chronic inhibition or chronic activation on behaviour.<br>For the CSS behaviour-photometry experiment, a sample size of 24 per group was used: this included a predicted 25% loss of subjects due to misplaced injection/optic fibre or inadequate signal, and allowance for possible high intra-group variance.<br>For the CSS basal amygdala-nucleus accumbens glutamate neuron transcriptome experiment, 20 CSS mice were included to allow for possible higher intra-group variance.
Data exclusions	In the CSS behaviour experiment, 1 (4%) mouse was excluded due to being a statistical outlier.<br>In the CSS behaviour-fibre photometry experiment, 5 mice (10%) were excluded due to no/low GCaMP6 signal as assessed prior to onset of behavioural testing, 4 mice (8%) due to technical failure, 1 mouse (2%) due to a misplaced optic fibre, and 3 mice (6%) due to being statistical outliers.<br>In the CSS basal amygdala-nucleus accumbens glutamate neuron transcriptome experiment, 7 mice (22%) were excluded due to insufficient CTB+ BA neurons at AP -1.6 to -2.0 mm, and 1 mouse (3%) was excluded due to being a statistical outlier following principal component analysis.<br>In the tetanus toxin light chain inhibition of basal amygdala-nucleus accumebns glutamate neurons experiment, 1 (3%) mouse was excluded due to misplaced BA injections.

Replication	The CSS effects on behaviour in the operant reward tests constitute replication. The previous findings have been published in several data papers. The experiments demonstrating CSS effects on basal amygdala-nucleus accumbens glutamate neuron activity during reward behaviour, and effects of basal amygdala-nucleus accumbens neuron inhibition or activation on reward behaviour, are novel findings and their replication has not yet been demonstrated.
Randomization	Pairs of mouse littermates were allocated to experimental groups semi-randomly but by taking into account their body weight and behavioural conditioning data, to ensure that the mean values of these parameters were counterbalanced across treatment groups.
Blinding	Because of the caging conditions it is not possible to conduct data collection for CSS experiments in a blinded manner. However, all data collection is automated. Blinding was introduced at the data processing level of fibre photometry data. Procedures for laser capture microdissection, RNA-sequencing and immunostaining were conducted blind to the identity of mouse treatment group.

Reporting for specific materials, systems and methods

We require information from authors about some types of materials, experimental systems and methods used in many studies. Here, indicate whether each material, system or method listed is relevant to your study. If you are not sure if a list item applies to your research, read the appropriate section before selecting a response.

Materials & experimental systems

n/a	Involved in the study
☐	☒ Antibodies
☒	☐ Eukaryotic cell lines
☒	☐ Palaeontology and archaeology
☐	☒ Animals and other organisms
☒	☐ Clinical data
☒	☐ Dual use research of concern

Methods

n/a	Involved in the study
☒	☐ ChIP-seq
☒	☐ Flow cytometry
☒	☐ MRI-based neuroimaging

Antibodies

Antibodies used	Primary antibodies:<br>Rabbit anti-VAMP2; Abcam, Ab3347<br>Rabbit anti-c-Fos; Cell Signalling, #2250<br>Secondary antibodies:<br>Cyanin5 goat anti-rabbit; Invitrogen, A10523
Validation	No in-house validation was conducted. Both primary antibodies have been widely used in many laboratories, and manufacturer provides details of validation.

Animals and other research organisms

Policy information about studies involving animals; ARRIVE guidelines recommended for reporting animal research, and Sex and Gender in Research

Laboratory animals	Mouse; strain C57BL/6RJ; bred in-house; males; aged 10 weeks at study onset<br>Mouse; strain CD-1; supplied by Janvier Labs France; males; aged 8-9 months
Wild animals	*Provide details on animals observed in or captured in the field; report species and age where possible. Describe how animals were caught and transported and what happened to captive animals after the study (if killed, explain why and describe method; if released, say where and when) OR state that the study did not involve wild animals.*
Reporting on sex	All experiments were conducted with male mice specifically. The chronic social stress protocol that is used is specific for males.
Field-collected samples	*For laboratory work with field-collected samples, describe all relevant parameters such as housing, maintenance, temperature, photoperiod and end-of-experiment protocol OR state that the study did not involve samples collected from the field.*
Ethics oversight	The experiments reported on were submitted in an application for an experimental license to the Veterinary Office, Canton of Zurich, a body completely independent of institutions conducting research. A license was issued, ZH155/2018, and the experiments were conducted under this license.

Note that full information on the approval of the study protocol must also be provided in the manuscript.
